# The sequence of cortical activity inferred by response latency variability in the human ventral pathway of face processing

**DOI:** 10.1038/s41598-018-23942-x

**Published:** 2018-04-11

**Authors:** Jo-Fu Lotus Lin, Juan Silva-Pereyra, Chih-Che Chou, Fa-Hsuan Lin

**Affiliations:** 1 Institute of Biomedical Engineering, National Taiwan University, Taipei, Taiwan; 2 Proyecto de Neurociencias, Facultad de Estudios Superiores Iztacala (FES-I), Universidad Nacional Autónoma de México, Tlalnepantla Estado de México, Mexico; 3 Integrated Brain Research Unit, Department of Medical Research, Taipei Veterans General Hospital, Taipei, Taiwan; 4 Department of Neuroscience and Biomedical Engineering, Aalto University, Espoo, Finland

## Abstract

Variability in neuronal response latency has been typically considered caused by random noise. Previous studies of single cells and large neuronal populations have shown that the temporal variability tends to increase along the visual pathway. Inspired by these previous studies, we hypothesized that functional areas at later stages in the visual pathway of face processing would have larger variability in the response latency. To test this hypothesis, we used magnetoencephalographic data collected when subjects were presented with images of human faces. Faces are known to elicit a sequence of activity from the primary visual cortex to the fusiform gyrus. Our results revealed that the fusiform gyrus showed larger variability in the response latency compared to the calcarine fissure. Dynamic and spectral analyses of the latency variability indicated that the response latency in the fusiform gyrus was more variable than in the calcarine fissure between 70 ms and 200 ms after the stimulus onset and between 4 Hz and 40 Hz, respectively. The sequential processing of face information from the calcarine sulcus to the fusiform sulcus was more reliably detected based on sizes of the response variability than instants of the maximal response peaks. With two areas in the ventral visual pathway, we show that the variability in response latency across brain areas can be used to infer the sequence of cortical activity.

## Introduction

Neural variability in response latency is usually considered to be caused by random noise. Based on the assumption that the measurement at each trial consists of an invariant response waveform and uncorrelated noise^1^, evoked responses are typically obtained via averaging across trials. The process of averaging effectively suppresses noise and increases the signal-to-noise ratio of the measurements. Because of averaging, response variability is often ignored.

The sequence of brain activity across areas has been typically inferred by the relative size of a specific feature of the evoked responses. Temporal features, such as onset or time-to-peak, have been widely used to estimate the progression of cortical activity. With magnetoencephalographic (MEG) measures, peak latencies from different cortical areas have been used to infer the sequence of activity. This strategy has been used to disclose sequential brain activity in studies of face perception^2^, reading^3,4^, online imitation of lip movements^5,6^, and somatosensory processing^7,8^. Peak latencies are typically defined as the interval between the stimulus onset and the time at which responses reach the maximal amplitude. However, measurements of peak latencies can be unstable and prone to noise contamination. Specifically, researchers have to decide to measure the latency of either positive or negative peaks, which may have similar magnitudes. Moreover, cortical responses may not always show clearly defined peaks. For example, multiple peaks are commonly present in the time courses in a given cortical area.

It has been shown that neural responses differ across trials when the identical stimulus is presented repeatedly: at the neuronal level, single-digit unit recordings suggested that neuronal responses to the identical visual stimuli varied considerably across trials in cat and monkey visual cortex^9–11^. It has also been reported that along the mammalian visual pathway, higher-order neurons, or neurons further away from sensory inputs, usually showed greater response variability in terms of spike timing^12,13^. The variability of neuronal responses could be due to noise from sensory or cellular levels, such as synaptic responses^10,14^. In addition, small amount of latency variability could accumulate as neural activity propagates or as information flows from lower to higher-level areas^12–14^. When large neuronal populations were recorded by non-invasive technique in healthy human brains, similar patterns of variability were found. For example, with a visuomotor task, Tang *et al*.^15^ found that the early MEG responses in the primary visual cortex showed smaller latency variability than the late responses.

Based on micro- and macro-scale neuronal findings in the visual cortex, we hypothesized that functional areas at later processing stages would have larger variability in response latency compared to areas at earlier processing stages. To test this hypothesis of increasing latency variability along the functional pathway, we examined if the size of regional latency variability would match the known sequence of cortical processing. Here, we used the ventral pathway of face processing to test this hypothesis.

Face-selective event-related fields or potentials measured by MEG or EEG (M170 or N170) have been reported previously^2,16,17^. The M170 or N170 response has been consistently localized to the fusiform gyrus^17–20^. Moreover, MEG studies have reported sequential activity from the calcarine fissure to the fusiform gyrus. Specifically, face-related cortical activity was found in the calcarine fissure with peak latencies around 110 ms followed by activity in the fusiform gyrus with peak latencies around 170 ms using either dipole models^17^ or distributed source estimates^21^. Taken together, the consensus is that the primary visual cortex functions as the initial stage to process general visual information, followed by the fusiform gyrus, which is specifically responsive to face detection and categorization^2,22^.

Based on the previous findings of sequential visual processing from the primary visual cortex to the fusiform gyrus, the goal of our present study was to characterize the variability of response latency during face processing. We specifically examined the variability of response latency in the primary visual area (the calcarine fissure) and the fusiform gyrus. Neural processing in these two areas was taken to reflect the sequence from lower-order primary sensory cortices to higher-order association areas. Using MEG measurements and based on our hypothesis, we expected that the variability of response latency in the calcarine fissure would be smaller than that in the fusiform gyrus. If true, the relative positions of cortical areas in the functional ventral pathway of face processing could be inferred by the size of response latency variability. Furthermore, we estimated the sequence of functional processing across brain areas in both the time and frequency domains by using dynamic and spectral decompositions of response latency variability. Dynamic estimates of response latency variability can reveal specific intervals of clear sequential processing, while spectral decomposition of response latency variability may disclose higher functional specificity as the neural activity at distinct oscillatory frequencies is related to different functions.

## Results

**Figure 1:**
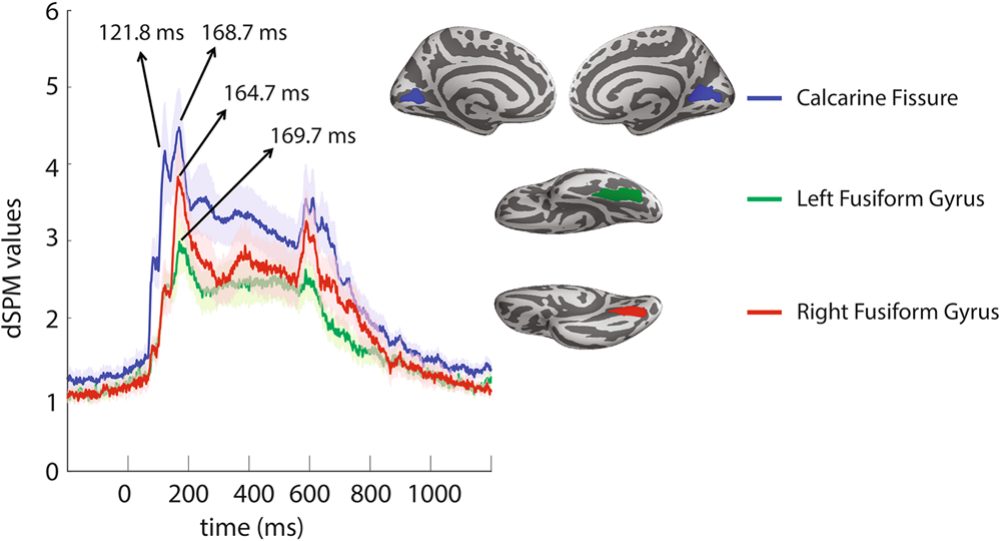
Group average time courses in the selected regions of interest in response to images of faces. Group average dynamic statistic parametric map (dSPM) values show response peaks in the calcarine fissure (blue), the left fusiform gyrus (green), and the right fusiform gyrus (red). Anatomically defined regions of interest are also shown. Shaded areas indicate the standard error of the mean across subjects.

Evoked responses to images of faces were observed in the calcarine fissure and fusiform gyrus in both the left and right hemispheres. Based on anatomically defined regions of interest (Fig. 1), the group average time courses showed two response peaks in the left calcarine fissure within the first 200 ms after the stimulus onset: the first peak at 121.8 ms and the second peak at 168.7 ms. The left fusiform gyrus showed a response peak at 169.7 ms. In the right hemisphere, two prominent response peaks were found at 121.8 ms and 167.7 ms in the calcarine fissure and one peak at 164.7 ms in the fusiform gyrus. Because no significant differences were observed between the time courses in the left and right calcarine fissure and we assumed no functional differences between the left and right calcarine fissures, time courses from the left and right calcarine fissures were combined for further analyses. Time courses combing the left and right calcarine fissure showed two peaks within the first 200 ms after the stimulus onset: at 121.8 ms and 168.7 ms.

**Figure 2:**
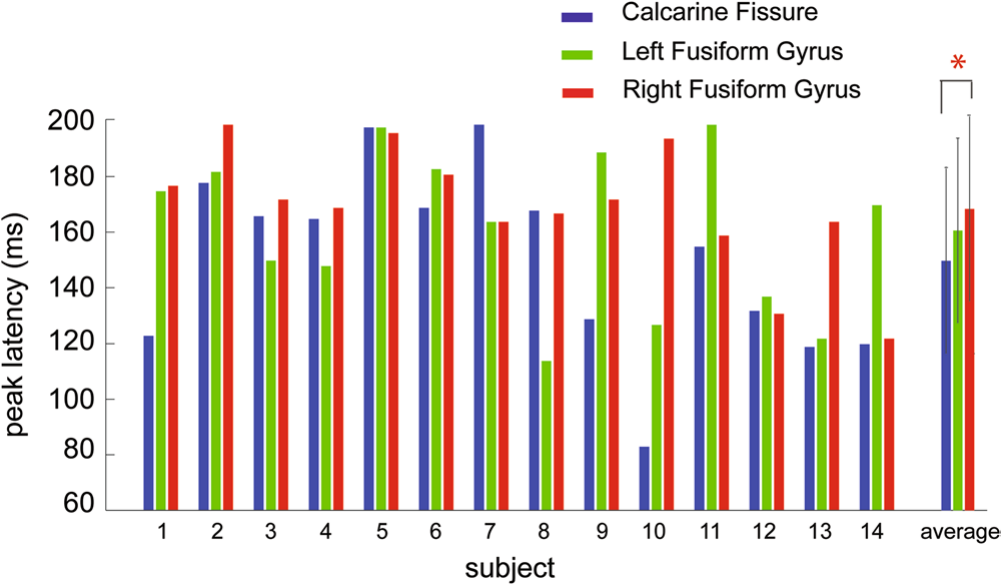
Peak latencies in the calcarine fissure, left fusiform gyrus, and right fusiform gyrus. Peak latencies, defined as the maximum amplitude reached during the time windows of interest from 70 to 200 ms, from each individual subject and the group averages are plotted. The whisks in the average represent the standard deviation of latency variability across subjects.

To compare peak latencies in the fusiform gyrus and the calcarine fissure, Wilcoxon signed rank test was used. By identifying the maximum peak latency within the pre-defined time window (70 ms–200 ms) from the average waveform in each cortical area from each subject separately (Fig. 2), we found that the peak latency in the calcarine fissure (150.0 ms+/−33.4 ms) was significantly earlier compared to that in the right fusiform gyrus (168.8 ms+/−22.0 ms; Z = 2.262, *p* = *0.024*). No significant difference was found between the peak latency in the calcarine fissure and the left fusiform gyrus (161.0 ms+/−28.4 ms; Z = 1.083, *p* = *0.279*). As shown in Fig. 2, nine out of 14 subjects showed earlier maximum peak latencies in the calcarine fissure than in the left fusiform gyrus. Ten out of 14 subjects showed earlier maximum peak latencies in the calcarine fissure than in the right fusiform gyrus. With regard to the response amplitude, the peak amplitude found in the calcarine fissure (5.4+/−2.4) was significantly stronger compared to that in the left fusiform gyrus (3.6+/−1.2; Z = −2.982, *p* = *0.003*), but not the right fusiform gyrus (4.5+/−1.4; Z = −1.538, *p* = *0.124*).

**Figure 3:**
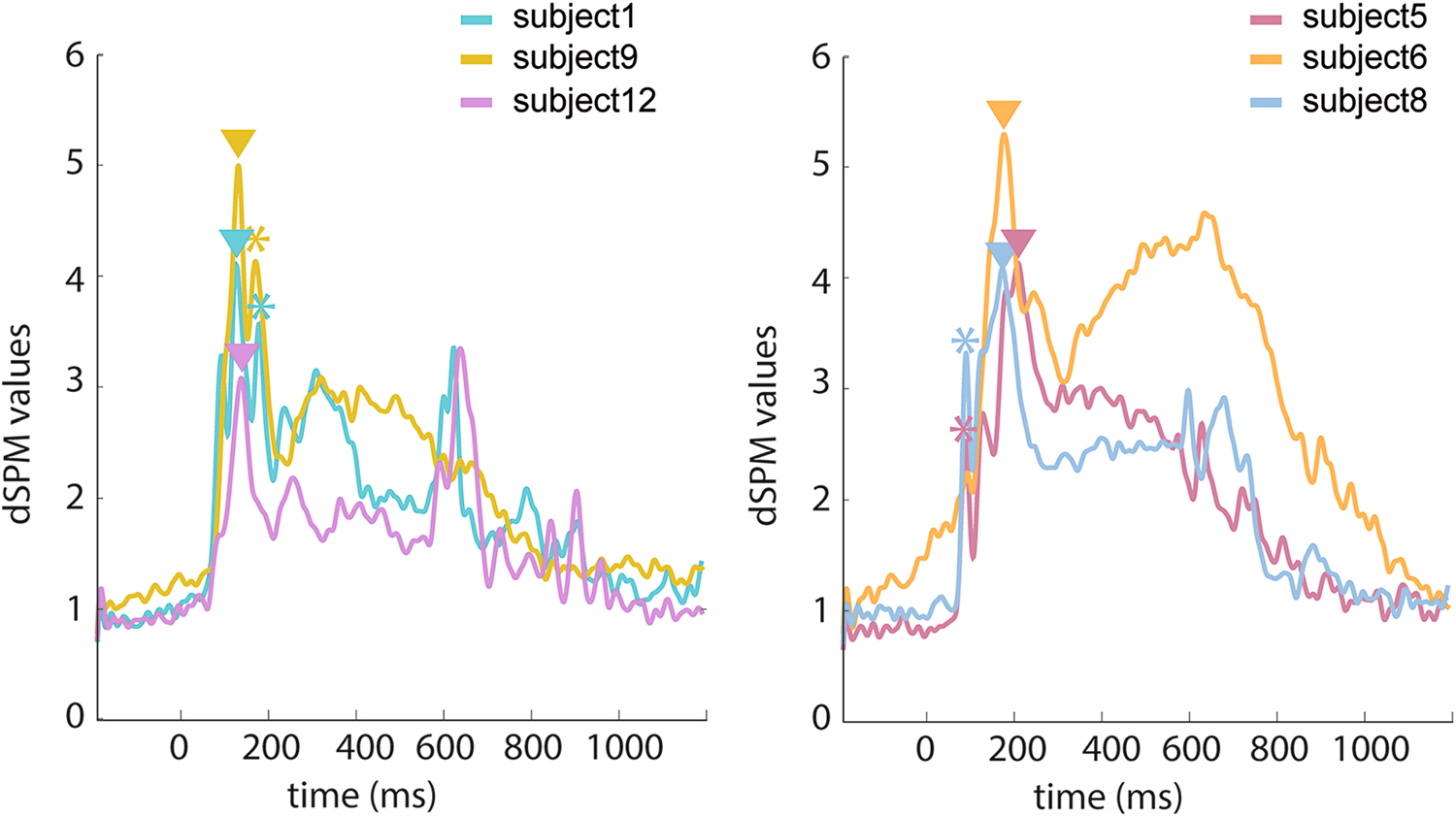
Time courses from individual subjects in the calcarine fissure. (Left) Waveforms from three representative subjects showing the maximum peak between 70 ms and 130 ms and a second smaller peak between140 ms and 200 ms. (Right) Waveforms from three other subjects showing the maximum peak between 140 ms and 200 ms and an earlier smaller peak between 70 ms and 130 ms. Triangles mark the maximum peaks and asterisks mark the second largest peaks between 70 ms and 200 ms in the time courses, respectively.

The left panel in Fig. 3 presents average waveforms from three representative subjects with the maximum peaks in the calcarine sulcus *before* 130 ms. Time courses from three other subjects are displayed in the right panel of Fig. 3, showing the maximum peaks in the calcarine sulcus *after* 130 ms. In the calcarine fissure, subjects with the maximum peak latency later than 130 ms contributed to the more delayed responses in our study than the responses reported in previous studies^2,21,23^. These results raised concerns about inferring the sequence of brain activity by the maximum peak latencies in related areas, which can vary across subjects considerably.

### Static estimates of latency variability

**Figure 4:**
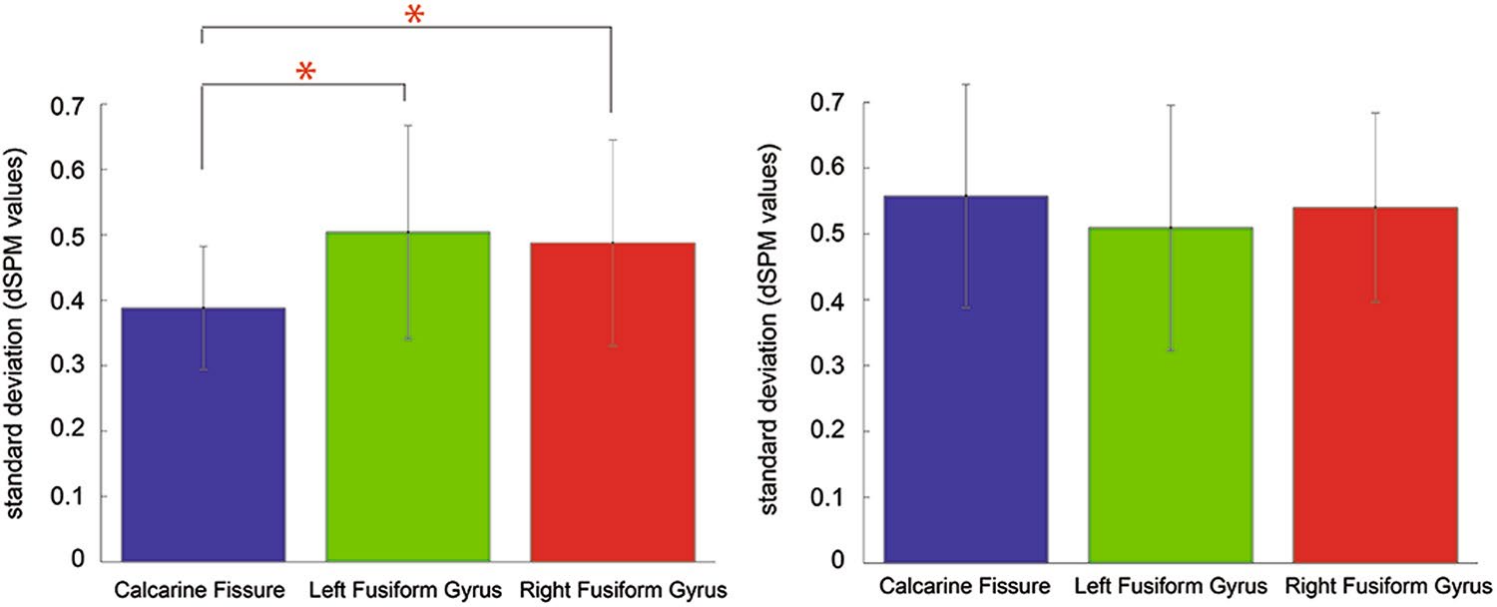
Noise levels of the baseline period across the three regions of interest. (Left) Noise levels were significantly smaller in the calcarine fissure compared to the left and right fusiform areas. (Right) After controlling for the noise levels, no significant differences in noise levels were found among ROIs.

Noise levels during the pre-stimulus baseline period were significantly larger in the left fusiform area compared to the calcarine fissure (Z = 3.045, *p* = *0.002*). Noise levels were also significantly higher in the right fusiform gyrus than the calcarine fissure (Z = 3.233, *p* = *0.001*) (Fig. 4, left). The difference in noise levels across regions could become a potential confounding factor in estimating latency variability. Thus, we added noise to equalize noise levels across areas (see Methods for details). As a result, the noise levels did not differ significantly between the left fusiform gyrus and the calcarine fissure (Z = −1.726, *p* = *0.084*), nor between the right fusiform gyrus and the calcarine fissure (Z = −1.350, *p* = *0.177*) (Fig. 4, right).

**Figure 5:**
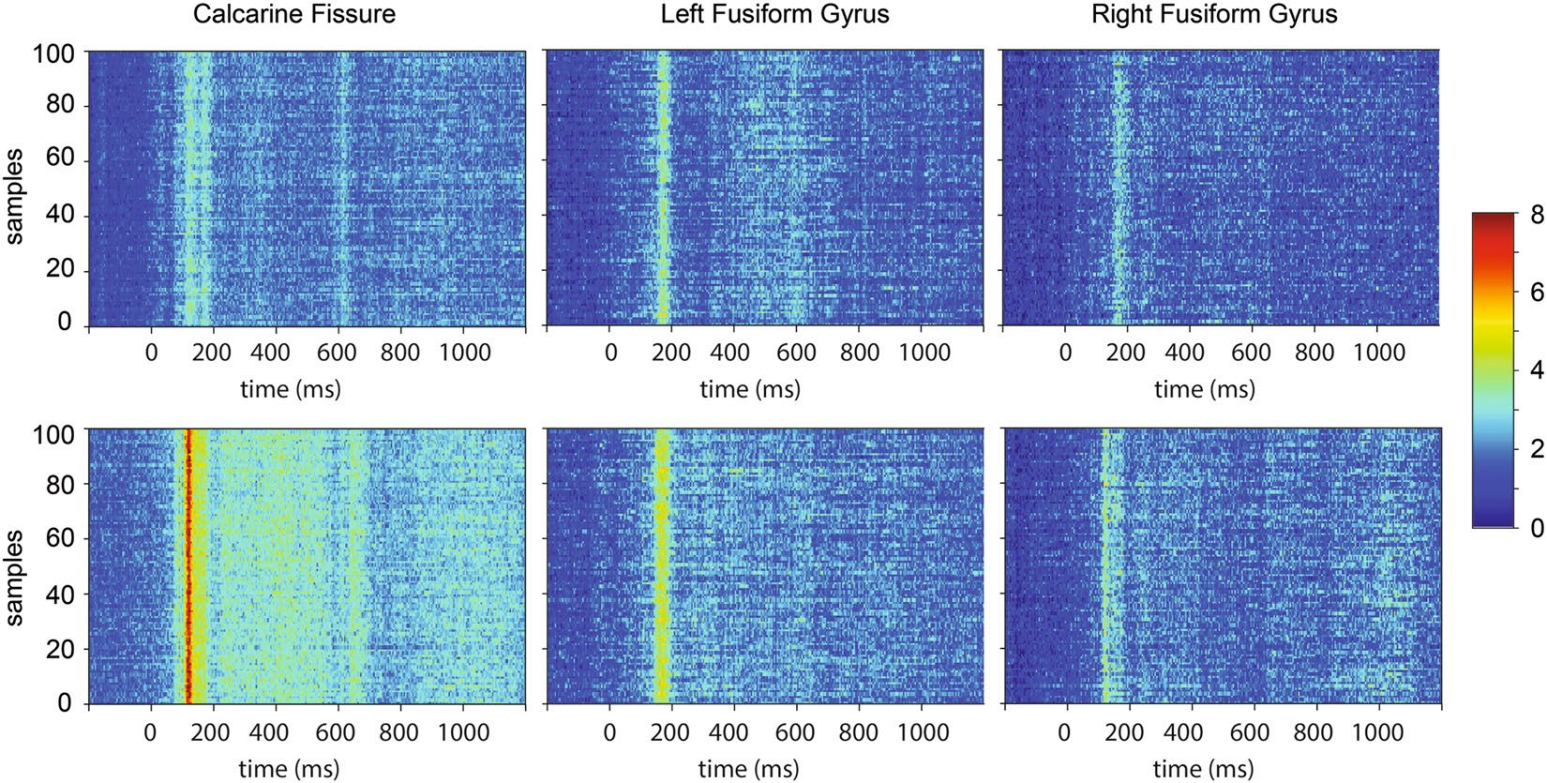
Bootstrap samples from the three regions of interest of two individual subjects. Each row in the data matrix represents one single bootstrap sample. Time 0 represents the time of stimulus onset. Coloring indicates dSPM values.

Because the signal-to-noise ratio of a single epoch is usually low, we used bootstrap samples to obtain a more robust estimate of relative latency. To ensure that our boostrap samples have a sufficient signal to noise ratio, we plotted evoked responses from each bootstrap sample in each region of interest. Figure 5 provides representative data from two individual subjects. A consistent pattern of stronger activity was observed between 100 and 200 ms after the stimulus onset across bootstrap samples.

**Figure 6:**
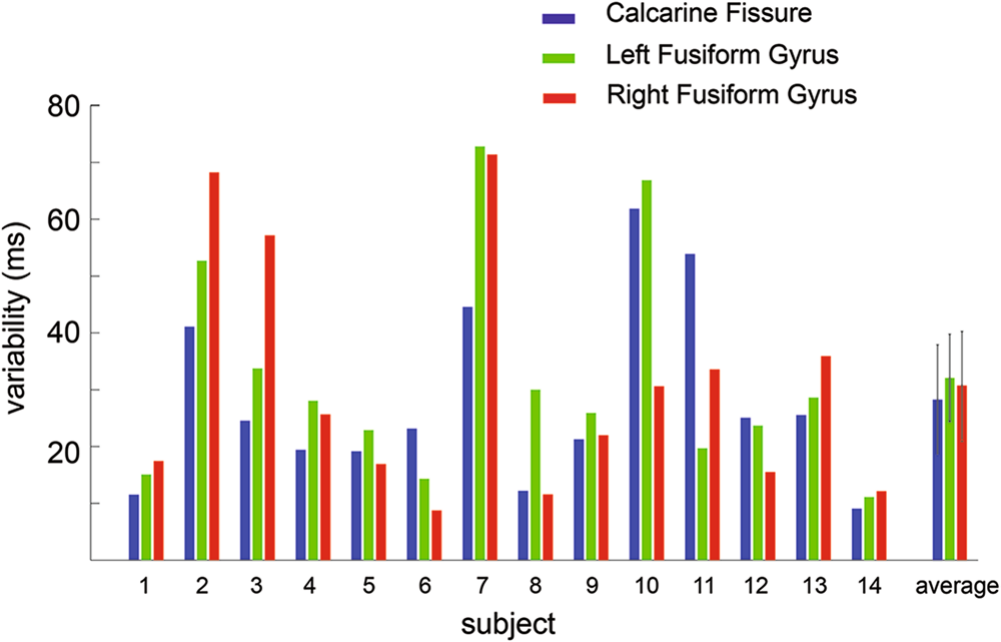
Intra-subject latency variability in the regions of interest during the presentation of faces. Intra-subject variability in the calcarine fissure (blue), left fusiform gyrus (green), and right fusiform gyrus (red) from each individual subject and the group averages are plotted. The whisks in the average represent the standard deviation of the variability across subjects.

For the static estimates of latency variability, we calculated the standard deviation of relative latencies across bootstrap samples. This latency variability was estimated for each subject and each area separately. We found that when face images were presented, there was a trend of larger latency variability in the left fusiform gyrus (31.8 ms+/−16.1 ms) than the calcarine fissure (28.0 ms+/−19.1 ms; Z = 1.790, *p* = *0.074*). Greater latency variability in the left fusiform gyrus compared to the calcarine fissure was found in 11 out of 14 subjects (Fig. 6). In addition, we found 8 out of 14 subjects showed larger latency variability in the right fusiform gyrus than in the calcarine fissure (Fig. 6). The latency variability of the right fusiform gyrus (30.5 ms+/−20.9 ms) was not significantly different from that of the calcarine fissure (28.0 ms+/−19.1 ms; Z = 0.471, *p* = *0.638*) across subjects.

**Figure 7:**
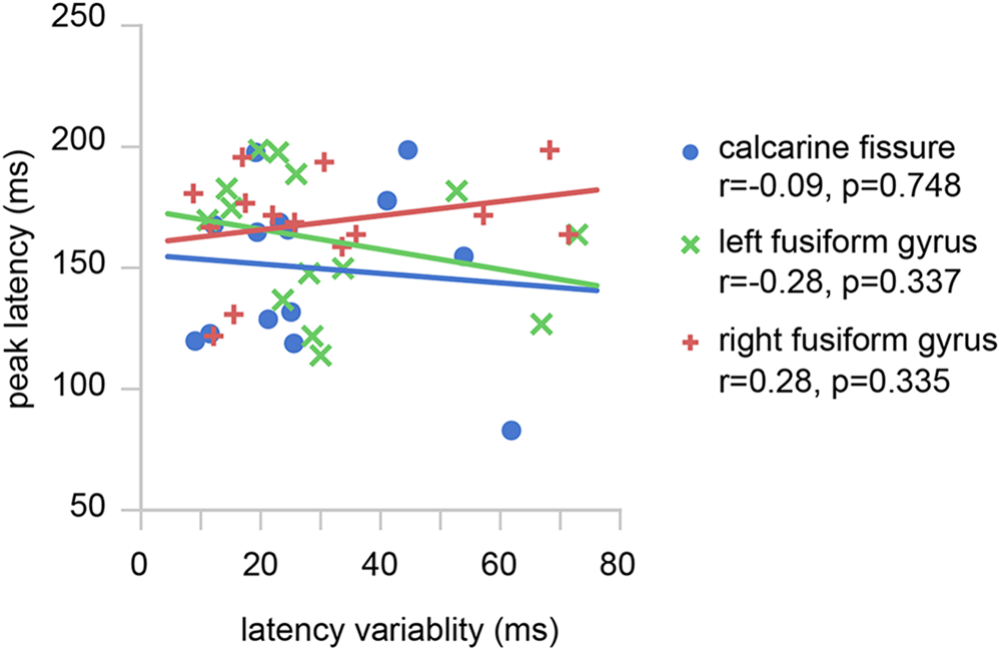
Correlations between the maximum peak latency and the latency variability in the calcarine fissure (blue), left fusiform gyrus (green), and right fusiform gyrus (red). No significant correlation between the maximum peak latency and response latency variability was found. Correlation coefficients (r’s) and *p*-values are also reported.

To test if the regional latency variability can be inferred from the maximum peak latency in regional responses, we correlated between these two quantities. No significant correlation was found in either the calcarine fissure, left fusiform gyrus, or right fusiform gyrus (Fig. 7). This result indicates that the latency variability in regional responses carries independent physiological information other than the maximum peak latency.

### Dynamic estimates of latency variability

**Figure 8:**
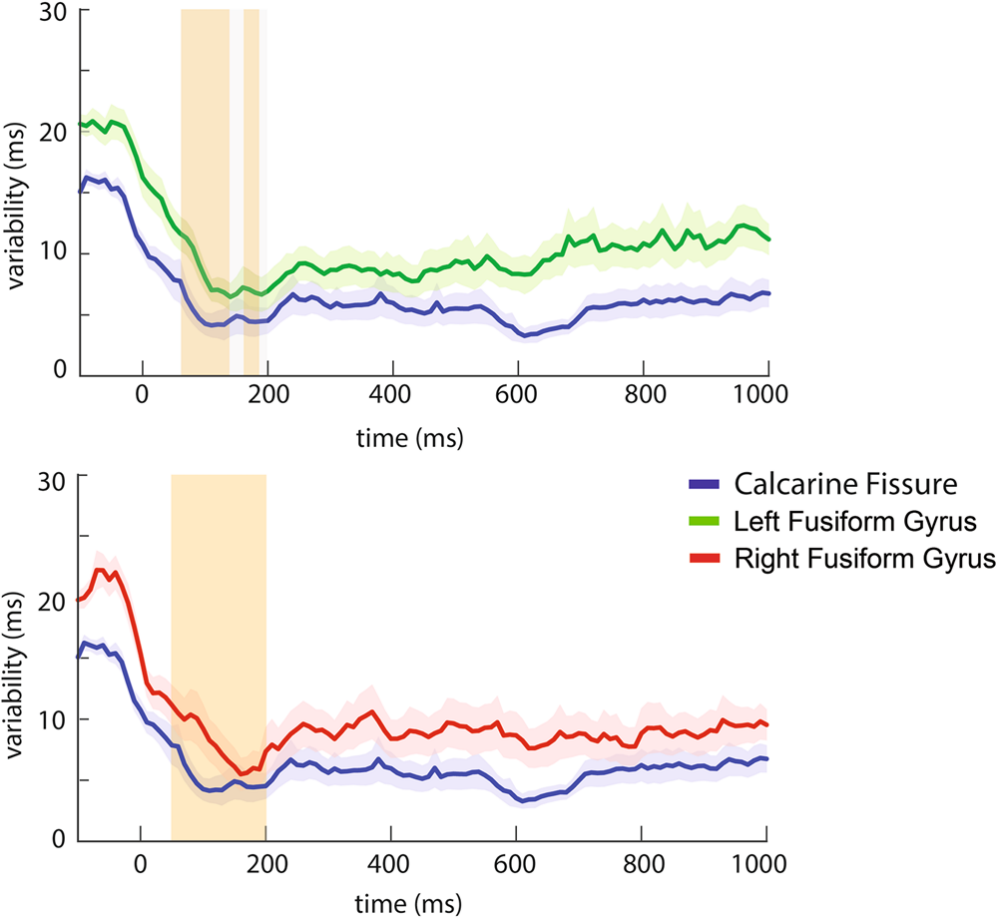
Latency variability across time windows in the temporal domain in the calcarine fissure compared to the left (top) and right fusiform gyrus (bottom). Shaded areas represent the standard error of the latency variability across subjects. Grey areas indicate time windows of interest from 70 to 200 ms. Vertical bars in orange indicate intervals showing significant differences in latency variability between cortical areas.

The latency variability was also estimated dynamically using a moving window approach. Dynamic estimates of latency variability are displayed in Fig. 8. Statistical comparisons were performed in the latency range between 70 ms and 200 ms. Significantly larger response latency variability was observed in the left fusiform gyrus than the calcarine fissure between 70 ms and 130 ms as well as between 170 ms and 180 ms (FDR *p* < 0.05). The right fusiform gyrus showed significantly larger response latency variability than the calcarine fissure between 70 ms and 200 ms (FDR *p* < 0.05).

To evaluate the sensitivity of different measures, we compared the number of subjects showing consistent patterns across regions of interest in term of the maximum peak latency and latency variability (Table 1). An earlier peak latency in the calcarine fissure than the fusiform gyrus would suggest a sequence of activity from the calcarine fissure to the fusiform gyrus. Smaller latency variability in the calcarine fissure compared to the fusiform gyrus would also suggest a sequence of activity from the calcarine fissure to the fusiform gyrus. However, differences between the tradition measures of maximum peak latency and the static measures of latency variability did not reach statistical significance (calcarine < left fusiform, Z = 0.837, *p* = *0.403*; calcarine < right fusiform, Z = −0.789, *p* = *0.430*). Differences between the traditional measures and the dynamic measures were also not statistically significant (calcarine < left fusiform, Z = 1.309, *p* = *0.190*; calcarine < right fusiform, Z = 1.480, *p* = *0.139*).

**Table 1:** Number of subjects showing earlier peak latency or smaller latency variability in the calcarine fissure compared to the left fusiform gyrus or the right fusiform gyrus.

		number of subjects/total
**maximum peak latency**	calcarine < left fusiform	9/14
	calcarine < right fusiform	10/14
**static variability**	calcarine < left fusiform	11/14
	calcarine < right fusiform	8/14
**dynamic variability**	calcarine < left fusiform	12/14
	calcarine < right fusiform	13/14

Percentages of subjects showing the above sequence of activity were also calculated. Averaging across the left and right fusiform gyrus, we found 68% of the tested subjects with earlier peak latencies in the calcarine fissure than the fusiform gyrus using the traditional measures of peak latency. Using the static estimates of latency variability, the percentage of subjects exhibiting smaller variability in the calcarine fissure compared to the fusiform gyrus was also 68%. Using the dynamic estimates of latency variability, 89% of the subjects showed smaller variability in the calcarine fissure compared to the fusiform gyrus. Overall, the dynamic estimates of latency variability showed a non-significant trend of higher sensitivity in detecting the specified sequence of activity, compared to the traditional measures of peak latency activity (Z = 1.382, *p* = *0.167*).

### Spectral estimates of latency variability

**Figure 9:**
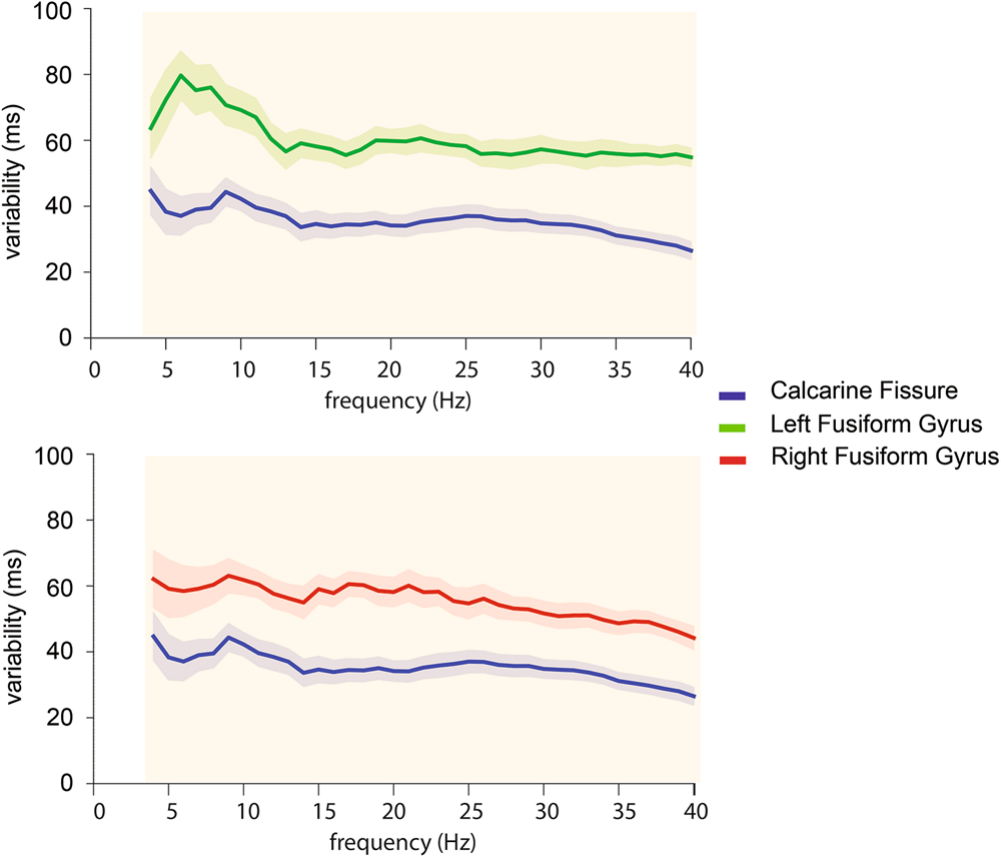
Latency variability across frequencies in the spectral domain in the calcarine fissure compared to the left (top) and right fusiform gyrus (bottom). Shaded areas represent the standard error of the latency variability across subjects. Vertical bars in orange indicate frequency ranges where significant differences between cortical areas in latency variability were found.

In the frequency domain, latency variability was estimated using spectrally decomposed time courses^24^. Estimated latency variability in the frequency domain is plotted in Fig. 9. Significantly larger variability was found in the left fusiform gyrus than the calcarine fissure at 4 to 40 Hz (FDR p < 0.05). The right fusiform gyrus showed significantly greater variability compared to the calcarine fissure at 4 to 40 Hz (FDR p < 0.05). Taken together, we found that most significant differences in the response variability were spectrally distributed between 4 and 40 Hz.

## Discussion

Our MEG results show that latency variability changes across cortical regions along the ventral pathway of face processing. The differential variability among regions was observed in both the temporal and spectral domains. Specifically, we found (1) significantly larger latency variability in the fusiform gyrus than the calcarine fissure between 70 to 130 ms and 170 to 180 ms in both hemispheres (Fig. 8). (2) In the frequency domain, significantly greater variability was observed in the fusiform gyrus than the calcarine fissure mostly between 4 and 40 Hz in both hemispheres (Fig. 9). In short, differences in the variability between functional areas studied here were found to reflect their relative positions in the ventral visual pathway: the earlier the processing stage, the smaller the response latency variability.

In our study, ROIs included the primary visual cortex as the region responsible for early visual processing and the fusiform gyrus as the region responsible for later face detection and identification. From the static estimates of latency variability, we found the fusiform gyrus, an area responsive to faces, showed a trend of greater latency variability than the calcarine fissure (Fig. 6).

While the static estimates of variability provide an overall measure of latency variability, the dynamic and spectral estimates of variability provide more temporally and spectrally specific analysis on latency variability by decomposing time series data into finer intervals and frequency bands. From the estimated latency variability in the temporal domain (Fig. 8), we found larger latency variability in the fusiform gyrus compared to the calcarine fissure. The increased latency variability from the calcarine fissure to the fusiform gyrus seems to reflect the temporal sequence of activity from the calcarine fissure to the fusiform gyrus as identified in previous studies^2,17,21^. These previous studies identified the maximum amplitudes from the sensor data or source data estimated using dipole models or distributed source estimates. Therefore, results from the present study support our postulate that the variability in regional response latency can serve to identify the sequence of activity.

Specifically, in the dynamic estimates, significantly larger variability was found in the fusiform gyrus compared to the calcarine fissure in the time window between 70 and 130 ms and 170 to 180 ms after the stimulus onset in both hemispheres. This latency range appears to be earlier than the typically reported face-selective evoked response around 170 ms^17,21^. Nevertheless, several studies have also reported face-selective responses start as early as 100 ms^2,25–28^. Taken together, these findings suggest that differences in variability in this early latency range can be associated with extracting stimulus information required to categorize faces or non-faces.

During the latency range between 70–200 ms, latency variability was relatively small compared to latency variability observed later than 200 ms. This pattern of response was observed in both the calcarine fissure and fusiform gyrus. Previous EEG studies have related increased signal variability to greater information transmission^29,30^. Hence, relatively large variability observed after 200 ms in our present study could indicate multiple sources of information exchange (e.g., feedforward and feeback signals), whereas small latency variability could be related to a simpler pattern of information inputs (e.g., feedforward signals only). When comparing latency variability across regions, significantly larger latency variability was found in the fusiform gyrus than the calcarine fissure in the latency range between 70–200 ms, but not after 200 ms. This result could reflect feedforward inputs or bottom-up effects from the calcarine fissure to the fusiform gyrus before 200 ms. However, in latency ranges later than 200 ms, both the feedforward and feedback signals may work at the same time. We speculate that such bidirectional information flows between the calcarine fissure and the fusiform gyrus could account for our finding that there were no dominant directions of influence and no significant differences in latency variability between the calcarine and fusiform gyri.

Our results also indicate that dynamic measures of latency variability could be more sensitive than static measures of latency variability (Table 1). By calculating the percentage of the tested subjects showing larger latency variability in the fusiform gyrus than the calcarine fissure, we found an improvement of about 20% using dynamic estimates compared to using static estimates of latency variability. Dynamic estimates of latency variability showed fluctuations of variability in the temporal domain. Because the feedforward signals seem transient, dynamic estimates using moving time windows could better characterize these transient changes and disclose sequential activity across cortical areas.

In the spectral domain, significantly different variability was found between 4 and 40 Hz (Fig. 9). Specifically, the latency variability was greater in the fusiform gyrus than the calcarine fissure across the theta, alpha, beta, and gamma bands. When comparing faces to non-face objects, previous ERP studies reported responses to faces in the theta, alpha, and gamma bands^31,32^ over posterior electrode positions. Using intracranial EEG, responses to faces were found in a wider frequency range, including the theta, alpha, beta, and gamma bands^33^. With MEG measures, significantly stronger responses in the right occipitotemporal area were reported between 4 and 25 Hz when comparing responses to faces and inverted faces^34^. These findings suggest that oscillatory activity across frequency bands could be associated with different aspects of face processing, such as information integration, face detection, or face identification. Taken together, the latency variability in frequency bands that have been associated with face processing is greater in the fusiform gyrus in both hemispheres than the calcarine fissure.

The latency variability observed here can be attributed to intrinsic variability of neural processing, such as synaptic transmission or neurotransmitter functions^10,14^. The intra-subject variability examined in the present study also suggests the possibility of intervening processes in local processing. These intervening processes could include fluctuations in top-down processes of attention or degrees of task engagement. To further validate our hypothesis of increasing latency variability as information flows across cortical regions, future studies should be conducted to test whether latency shifts remain constant along different functional pathways, or increase as the cortical area gets more distant from primary sensory processing.

The results from our present study indicate that there are no significant correlations between the static estimates of latency variability and the traditional measures of maximum peak latency (Fig. 7). In addition, the number of subjects showing larger peak latency or latency variability in the fusiform gyrus than the calcarine fissure tends to increase from the traditional measure to the dynamic measures (Table 1). The dynamic measures of latency variability are more sensitive to detect the sequence of activity than peak latencies defined within pre-determined time windows, suggesting that our method is more generalized in analyzing sequential processing in other experiments involving areas subserving different functions.

Our results also reveal that peak latency might not be the optimal index to estimate the order of regional activity, particularly when the variability of regional response latency is large, as exemplified by our analysis (Fig. 3). Averaging regional response waveforms smears out peaks and reduces the peak amplitude. Large regional response variability may thus result in no explicit response peaks in the average waveform. Moreover, when prior knowledge of response time courses is not well known, the dynamic and spectral estimates of latency variability without specifying a time window can provide more sensitive measures than using peak latencies within pre-defined time windows as we demonstrated here (Table 1). When response time courses are unknown, selecting a wider time window can be the first analysis approach. However, by using a wide latency window, peak latencies may be shifted to later latencies when multiple peaks are present in the time course as shown in our data. For estimating the processing pipeline in the brain, methods other than finding peak latencies, such as finding relative latencies via the cross-correlation method implemented in our study, may be more robust.

One approach of detecting regional information flow is Granger causality^35–37^. However, Granger causality assumes the time series should be stationary. This assumption is seriously challenged in evoked brain responses. For example, our dynamic estimates of variability indicate latency variability does not remain constant over time. It should be noted that while our approach does not require the stationary assumption and can be used to estimate the relative positions of regional processing in a functional pathway, we did not infer any causal influence across brain regions.

In our analysis, the cross-correlation procedure requires a template waveform. Here, we used the averaged waveform across trials as the template. Because the template is defined for each subject and each ROI separately, variations between individuals do not exert influence in our analysis. However, variations within subjects or across trials still remains. Furthermore, our bootstrap samples were not single-trial responses, but averaged across a subsample of trials. The number of trials chosen to generate one bootstrap sample can affect our estimates of variability and the signal-to-noise ratio. We found that the sensitivity of detecting the specified sequence of activity across subjects was stable when using between 20 to 40 trials to form one bootstrap sample (see Supplementary Information for details). Cautions should be taken to determine the number of trials in generating the bootstrap samples in order to minimize the noise fluctuation and to detect the response variability at the same time.

## Conclusions

Using images of human faces to elicit neuromagnetic responses in brain areas subserving visual processing, we found that the order of brain areas demonstrating progressively larger response latency variability matched the expected sequence of information transfer across brain areas. Our present study suggests that regional response latency variability can provide meaningful information about the underlying sequence of neural processes. Differential variability between cortical areas can reflect their relative positions in a functional pathway. The close correspondence between temporal variability and the sequence of cortical activity in other functional processing pathways should be further tested with other complex stimuli and tasks.

## Materials and Methods

### Subjects

Fourteen subjects (mean age: 27.9+/−6.2 years; 8 females and 6 males) participated in this study. All subjects provided written inform consent prior to participation and the experimental protocols were approved by the Institute of Review Board at the National Taiwan University Hospital. All experiments were carried out in accordance with the approved guidelines. All subjects had normal or corrected to normal vision.

### Stimuli and Tasks

Visual stimuli of neutral faces from eight individuals^38,39^ and the phase-scrambled versions of the same stimuli were presented randomly to subjects. Images of faces or scrambled faces were presented for 500 ms in duration with a random inter-stimulus interval ranging between 1800 and 2200 ms. Stimuli were presented using Presentation (Version 18.0, Neurobehavioral Systems, Inc.). To ensure that subjects were attending to the visual stimuli, subjects were instructed to press a button when two sequential images were identical. Four runs (about 9 minutes per run) of data were collected from each subject. About 200 trials of faces and scrambled faces were shown to each subject in each run.

### MEG data acquisition and preprocessing

MEG data were recorded using a 306-channel whole-head MEG system (VectorView, Elekta-NeuroMag Oy, Helsinki, Finland). MEG signals were band-pass filtered between 0.03–200 Hz and digitized at 1000 Hz. Temporal signal space separation^40,41^ was not used. Epochs with EOG exceeding 150 uV and MEG exceeding 3 pT/cm (gradiometers) and 4 pT (magnetometers) were removed from offline averages to suppress artifacts related to eye movements and system instability.

### MRI data acquisition and processing

Anatomical data were measured on a 3 T MR scanner (Tim Trio, Siemens, Erlangen, Germany) with a 32-channel head coil array. Three-dimensional *T*_1_-weighted structural images were collected (MP-RAGE sequence: TR = 2530 ms, TE = 3.03 ms, TI = 1100 ms, flip angle = 7°, slice thickness = 1 mm, image matrix = 256 × 224, voxel size = 1 × 1 × 1 mm^3^). For visualization and localizing anatomical landmarks, cortical surfaces were reconstructed using FreeSurfer (version 5.1.0; http://surfer.nmr.mgh.harvard.edu).

### MEG source analysis

MEG sources were estimated using anatomically constrained minimum-norm estimates (MNE)^42,43^ on the cortical surface. The MEG source spaces had about 7,000 vertices on each hemisphere. The forward models were calculated using Boundary Element Method (BEM) based on *T*_1_-weighted MRI data^44^. MEG noise covariance matrices were calculated using the 200 ms baseline prior to the stimulus presentation. Noise-normalized MNE, also called dynamic statistical parametric maps (dSPM)^42^, was calculated at each time point to map the extracranial MEG data onto cortical surfaces. Before averaging across subjects, source estimates from each individual subject were morphed to an average brain provided by FreeSurfer (subject “fsaverage”)^45,46^.

Four regions of interest (ROIs) were defined by anatomical labels^47^. These four ROIs were the calcarine fissure and fusiform gyrus in the left and right hemispheres. In this study, the calcarine fissure is taken as the low-order visual area, processing sensory information before the information is sent to higher-level areas. The fusiform gyrus, part of the core system of face processing, is taken as the high-order visual areas for detecting and identifying faces^22,48^.

For comparisons, we also followed the conventional method of using peak latencies across regions to estimate the sequence of activity. In previous MEG studies when conditions of face and non-face were compared^17,21,23^, the maximum peak latency was defined as the time when the average response amplitude reached its maximum within the time intervals of interest. For example, the time intervals of interest were defined between +/−30 ms of the M100 responses in the calcarine fissure and M170 responses in the fusiform gyrus; that is, between 70 ms and 130 ms for the calcarine fissure and between 140 ms and 200 ms for the fusiform gyrus. However, these definitions already assumed an earlier response window for activity in the calcarine fissure compared to the fusiform gyrus. Defining the time windows of interest differently for the two regions of interest would defeat our purpose of identifying sequences of activity across regions. Thus, we used the same time window of interest for both ROIs. Our time window of interest was defined between 70 ms and 200 ms, wide enough to cover the activity in both the calcarine fissure and the fusiform gyrus.

### Static estimates of latency variability

To estimate relative latency, we first calculated template waveforms separately for each subject in each ROI (the calcarine fissure and fusiform gyrus bilaterally). The template waveform was obtained by averaging dSPM source waveforms across trials and across vertices within a given ROI. To cope with the low signal-to-noise ratio from single-epoch waveform, we estimated evoked responses using a bootstrap approach. Specifically, we obtained one bootstrap sample by randomly selecting 30 single-trial source estimates (approximately 15% of the trials) and averaging over these trials. The bootstrap procedure was repeated 100 times for each condition, ROI, and subject. These responses were then low-pass filtered at 40 Hz.

We also compared noise levels across ROIs. Noise levels were estimated by the standard deviation over the pre-stimulus or baseline period (−200 to 0 ms) and averaged across bootstrap samples and across subjects. To avoid potential confounds caused by regional differences in noise levels, we equalized noise levels across regions by adding Gaussian random noise (mean = 0 and standard deviation = differences in noise levels between regions) to the bootstrap samples across time.

Then, we calculated cross-correlations between the template waveform and each bootstrap sample in each ROI. The template waveform was temporally shifted (−200 and +200 ms in steps of 10 ms). The shift corresponding to the maximum correlation coefficient was defined as the relative latency. With 100 relative latencies across bootstrap samples, we calculated intra-subject latency variability or the standard deviation of relative latencies across bootstrap samples. Intra-subject latency variability in the calcarine fissure and fusiform gyrus was compared using Wilcoxon signed-rank test.

To further understand the relationship between the static estimates of latency variability and the traditional measures of peak latency, Pearson’s correlation coefficients were calculated between the static estimates of latency variability and the peak latency obtained using the traditional method as described in the previous section.

### Dynamic estimates of latency variability

Dynamic latency variability was calculated by first convolving the template waveform and each bootstrap sample with a Gaussian window with standard deviation of 30 ms. The window center was temporally shifted across the entire epoch, from 100 ms before the stimulus onset to 1000 ms after the stimulus onset in increments of 10 ms. Relative latencies were calculated using the same cross-correlation method as described above. Intra-subject latency variability was obtained by calculating the standard deviation of relative latencies across bootstrap samples within each time window. Within the pre-defined time intervals from 70 to 200 ms, the Wilcoxon signed-rank test was then used to compare intra-subject latency variability in the calcarine fissure and fusiform gyrus in each time window. Multiple tests over latency windows were controlled by the false discovery rate (FDR)^49^.

To further compare the sensitivity of different measures (i.e., the traditional measures of peak latency, static estimates, and dynamic estimates of latency variability), we counted the number of subjects showing a sequence of activity from the calcarine fissure to the fusiform gyrus from each measure. With the traditional measures of peak latency, a smaller peak latency in the calcarine fissure compared to the fusiform gyrus would suggest a sequence of activity from the calcarine fissure to the fusiform gyrus. With the static and dynamic estimates of latency variability, greater latency variability in the fusiform gyrus compared to the calcarine fissure would indicate a sequence of activity from the calcarine fissure to the fusiform gyrus. For the dynamic estimates of latency variability, the number of subjects showing the above sequence of activity was averaged across time windows and then rounded up or down to the nearest integer. To test whether the proportions or numbers of subjects differ between measures, Z-test was used.

### Spectral estimates of latency variability

In the frequency domain, latency variability was obtained by first calculating the Temporal Spectral Evolution (TSE)^24^ for the template and bootstrap samples. TSE was calculated by convolving time series data with a Morlet wavelet^50^ with width = 5. The center frequency of wavelet varied from 4 Hz to 40 Hz in steps of 1 Hz. The spectral power time series was then used for calculating relative latency and latency variability using the cross-correlation method described above. Intra-subject variability in latency between the calcarine fissure and the fusiform gyrus was compared using the Wilcoxon signed-rank test at each frequency. Multiple comparisons across frequencies were controlled by FDR^49^. All calculations were done using Matlab (Mathworks, Natick, MA, USA).

## Electronic supplementary material

Supplementary Information

## References

[1] Dawson GD (1954). A multiple scalp electrode for plotting evoked potentials. Electroencephalogr Clin Neurophysiol.

[2] Liu J, Harris A, Kanwisher N (2002). Stages of processing in face perception: an MEG study. Nat Neurosci.

[3] Salmelin R, Helenius P, Service E (2000). Neurophysiology of fluent and impaired reading: a magnetoencephalographic approach. J Clin Neurophysiol.

[4] Vartiainen J, Liljestrom M, Koskinen M, Renvall H, Salmelin R (2011). Functional magnetic resonance imaging blood oxygenation level-dependent signal and magnetoencephalography evoked responses yield different neural functionality in reading. J Neurosci.

[5] Nishitani N, Avikainen S, Hari R (2004). Abnormal imitation-related cortical activation sequences in Asperger’s syndrome. Ann Neurol.

[6] Nishitani N, Hari R (2002). Viewing lip forms: cortical dynamics. Neuron.

[7] Korvenoja A (1999). Activation of multiple cortical areas in response to somatosensory stimulation: combined magnetoencephalographic and functional magnetic resonance imaging. Hum Brain Mapp.

[8] Ioannides AA (2002). Timing and connectivity in the human somatosensory cortex from single trial mass electrical activity. Hum Brain Mapp.

[9] Tomko GJ, Crapper DR (1974). Neuronal variability: non-stationary responses to identical visual stimuli. Brain Res.

[10] McAdams CJ, Maunsell JH (1999). Effects of attention on the reliability of individual neurons in monkey visual cortex. Neuron.

[11] Tolhurst DJ, Movshon JA, Dean AF (1983). The statistical reliability of signals in single neurons in cat and monkey visual cortex. Vision Res.

[12] Bair W (1999). Spike timing in the mammalian visual system. Curr Opin Neurobiol.

[13] Tiesinga P, Fellous JM, Sejnowski TJ (2008). Regulation of spike timing in visual cortical circuits. Nat Rev Neurosci.

[14] Faisal AA, Selen LP, Wolpert DM (2008). Noise in the nervous system. Nat Rev Neurosci.

[15] Tang, A. C., Pearlmutter, B. A., Zibulevsky, M., Hely, T. A. & Weisend, M. P. An MEG study of response latency and variability in the human visual system during a visual-motor integration task. *Advances in Neural Information Processing Systems*., 185–191 (1999).

[16] Bentin S, Allison T, Puce A, Perez E, McCarthy G (1996). Electrophysiological Studies of Face Perception in Humans. J Cogn Neurosci.

[17] Halgren E, Raij T, Marinkovic K, Jousmaki V, Hari R (2000). Cognitive response profile of the human fusiform face area as determined by MEG. Cereb Cortex.

[18] Deffke I (2007). MEG/EEG sources of the 170-ms response to faces are co-localized in the fusiform gyrus. Neuroimage.

[19] Linkenkaer-Hansen K (1998). Face-selective processing in human extrastriate cortex around 120 ms after stimulus onset revealed by magneto- and electroencephalography. Neurosci Lett.

[20] Watanabe S, Miki K, Kakigi R (2005). Mechanisms of face perception in humans: a magneto- and electro-encephalographic study. Neuropathology.

[21] Marinkovic K, Courtney MG, Witzel T, Dale AM, Halgren E (2014). Spatio-temporal dynamics and laterality effects of face inversion, feature presence and configuration, and face outline. Front Hum Neurosci.

[22] Grill-Spector K, Knouf N, Kanwisher N (2004). The fusiform face area subserves face perception, not generic within-category identification. Nat Neurosci.

[23] Sams M, Hietanen JK, Hari R, Ilmoniemi RJ, Lounasmaa OV (1997). Face-specific responses from the human inferior occipito-temporal cortex. Neuroscience.

[24] Salmelin R, Hari R (1994). Spatiotemporal characteristics of sensorimotor neuromagnetic rhythms related to thumb movement. Neuroscience.

[25] Dering B, Martin CD, Moro S, Pegna AJ, Thierry G (2011). Face-sensitive processes one hundred milliseconds after picture onset. Front Hum Neurosci.

[26] Herrmann MJ, Ehlis AC, Ellgring H, Fallgatter AJ (2005). Early stages (P100) of face perception in humans as measured with event-related potentials (ERPs). J. Neural Transm.

[27] Itier RJ, Taylor MJ (2004). N170 or N1? Spatiotemporal differences between object and face processing using ERPs. Cereb Cortex.

[28] Colombatto C, McCarthy G (2017). The Effects of Face Inversion and Face Race on the P100 ERP. J Cogn Neurosci.

[29] Misic B, Vakorin VA, Paus T, McIntosh AR (2011). Functional embedding predicts the variability of neural activity. Front Syst Neurosci.

[30] Vakorin VA, Lippe S, McIntosh AR (2011). Variability of brain signals processed locally transforms into higher connectivity with brain development. J Neurosci.

[31] Rousselet GA, Husk JS, Bennett PJ, Sekuler AB (2007). Single-trial EEG dynamics of object and face visual processing. Neuroimage.

[32] Zion-Golumbic E, Bentin S (2007). Dissociated neural mechanisms for face detection and configural encoding: evidence from N170 and induced gamma-band oscillation effects. Cereb Cortex.

[33] Klopp J, Halgren E, Marinkovic K, Nenov V (1999). Face-selective spectral changes in the human fusiform gyrus. Clin Neurophysiol.

[34] Hsiao FJ (2006). Oscillatory characteristics of face-evoked neuromagnetic responses. Int J Psychophysiol.

[35] Deshpande G, Sathian K, Hu X (2010). Effect of hemodynamic variability on Granger causality analysis of fMRI. Neuroimage.

[36] Granger CWJ (1969). Investigating causal relations by econometric models and cross-spectral methods. Econometrica.

[37] Roebroeck A, Formisano E, Goebel R (2005). Mapping directed influence over the brain using Granger causality and fMRI. Neuroimage.

[38] Ekman P (1992). Are there basic emotions?. Psychol Rev.

[39] Woods, D. L., Yund, E. W. & Kang, X. J. Unified functional/anatomical maps of human auditory cortex. *Archives of Neurobehavioral Experiments and Stimuli*, 46 (2003).

[40] Taulu S, Simola J (2006). Spatiotemporal signal space separation method for rejecting nearby interference in MEG measurements. Phys Med Biol.

[41] Taulu S, Hari R (2009). Removal of magnetoencephalographic artifacts with temporal signal-space separation: demonstration with single-trial auditory-evoked responses. Hum Brain Mapp.

[42] Dale AM (2000). Dynamic statistical parametric mapping: combining fMRI and MEG for high-resolution imaging of cortical activity. Neuron.

[43] Dale AM, Sereno MI (1993). Improved localization of cortical activity by combining EEG and MEG with MRI cortical surface reconstruction: a linear approach. J Cogn Neurosci.

[44] Hämäläinen MS, Sarvas J (1989). Realistic conductivity geometry model of the human head for interpretation of neuromagnetic data. IEEE Trans Biomed Eng.

[45] Dale AM, Fischl B, Sereno MI (1999). Cortical surface-based analysis. I. Segmentation and surface reconstruction. Neuroimage.

[46] Fischl B, Sereno MI, Dale AM (1999). Cortical surface-based analysis. II: Inflation, flattening, and a surface-based coordinate system. Neuroimage.

[47] Destrieux C, Fischl B, Dale A, Halgren E (2010). Automatic parcellation of human cortical gyri and sulci using standard anatomical nomenclature. NeuroImage.

[48] Haxby JV, Hoffman EA, Gobbini MI (2000). The distributed human neural system for face perception. Trends Cogn Sci.

[49] Nichols T, Hayasaka S (2003). Controlling the familywise error rate in functional neuroimaging: a comparative review. Stat Methods Med Res.

[50] Tallon-Baudry C, Bertrand O (1999). Oscillatory gamma activity in humans and its role in object representation. Trends Cogn Sci.

