# Supplementary material for: The sequence of cortical activity inferred by response latency variability in the human ventral pathway of face processing
Source: Sci Rep. 2018 Apr 11;8:5836. doi: 10.1038/s41598-018-23942-x (PMC5895585; doi:10.1038/s41598-018-23942-x)
Supplement: Supplementary file 1 — Supplementary Information [file 41598_2018_23942_MOESM1_ESM.pdf]

The sequence of cortical activity inferred by response latency variability in the human ventral pathway of face processing

Jo-Fu Lotus Lin[1], Juan Silva-Pereyra[2], Chih-Che Chou[1,3], Fa-Hsuan Lin[1,4]

[1]Institute of Biomedical Engineering, National Taiwan University, Taipei, Taiwan
[2]Proyecto de Neurociencias, Facultad de Estudios Superiores Iztacala (FES-I), Universidad Nacional Autónoma de México, Tlalnepantla Estado de México, México
[3]Integrated Brain Research Unit, Department of Medical Research, Taipei Veterans General Hospital, Taipei, Taiwan
[4]Department of Neuroscience and Biomedical Engineering, Aalto University, Espoo, Finland

Supplementary Information

Effects of the number of trials included in one bootstrap sample

Methods

In our analysis, we randomly selected *m* trials from all *n* trials collected. From the selected *m* trials, we calculated the average response to form a single bootstrap sample. Thus, a choice of *m* had to be made. When choosing *m*, we should consider the traded-off between capturing the variability (preferring fewer trials) and increasing the signal-to-noise ratio (preferring more trials).

To investigate how *m*, or the number of trials used for one bootstrap sample, affects the detection of a response sequence (from the calcarine fissure to the fusiform gyrus), we ran our analysis with different *m* values. Specifically, we varied *m* from 10 to 100. For each *m*, we calculated the static, dynamic, and spectral estimates of latency variability (**Figure S1 left**). For the dynamic and spectral estimates of latency variability, we averaged latency variability across time windows (70 to 200

ms) and frequency ranges (4 to 40 Hz), respectively. The time window and the frequency range were chosen based on our findings showing significant differences when $m = 30$ (as shown in **Figure 6, Figure 8**, and **Figure 9**). Therefore, examining results of different m values can directly test if our variability estimation with m = 30 was stable. In addition, we calculated the proportion of subjects showing the expected activity sequence (**Figure S1 right**). The expected sequence was larger latency variability in the fusiform gyrus than in the calcarine fissure, suggesting a sequence of activity from the calcarine fissure to the fusiform gyrus.

Results

For the static estimates of latency variability, larger variability in the fusiform gyrus compared to the calcarine fissure was consistently observed across different m values (**Figure S1a left**). The difference in the latency variability between the fusiform gyrus and calcarine fissure decreased when m increased. However, the number of subjects showing the expected response pattern (variability: calcarine < fusiform) did not differ significantly between 20 and 30 trials (calcarine < left fusiform, Z = 0.493, $p = 0.622$; calcarine < right fusiform, Z = 0.0, $p = 1.0$), nor between 30 and 40 trials (calcarine < left fusiform, Z = 0.0, $p = 1.0$; calcarine < right fusiform, Z = 0.0, $p = 1.0$) (**Figure S1a right**). Thus, results were consistent when we used an m of 20, 30, or 40 trials.

For the dynamic and spectral estimates of latency variability, higher variability in the fusiform gyrus compared to the calcarine fissure was consistently observed across different m values (**Figure S1b and Figure S1c left**). The difference in the latency variability between the fusiform gyrus and the calcarine fissure decreased as m increased. However, when comparing the number of subjects showing the expected response pattern (variability: calcarine < fusiform) with 20 and 30 trials in the dynamic variability estimates, no significant differences were found (calcarine < left fusiform, Z = 0.0, $p = 1.0$; calcarine < right fusiform, Z = 0.0, $p = 1.0$). Also, there were no significant differences between 30 and 40 trials (calcarine < left fusiform, Z = 0.0, $p = 1.0$; calcarine < right fusiform, Z = 0.0, $p = 1.0$) (**Figure S1b right**). For the

spectral estimates, no significant differences were found between 20 and 30 trials (calcarine < left fusiform, Z = 0.0, *p = 1.0*; calcarine < right fusiform, Z = 0.611, *p = 0.541)*. There were also no significant differences between 30 and 40 trials (calcarine < left fusiform, Z = 0.611, *p = 0.541*; calcarine < right fusiform, Z = 0.611, *p = 0.541)* (**Figure S1c right**). That is, using an *m* of 20, 30, or 40 trials gave consistent results in the dynamic and spectral estimates of the latency variability.

In summary, the detection of a response sequence (from the calcarine fissure to the fusiform gyrus) was stable across different *m* values in estimating the latency variability.

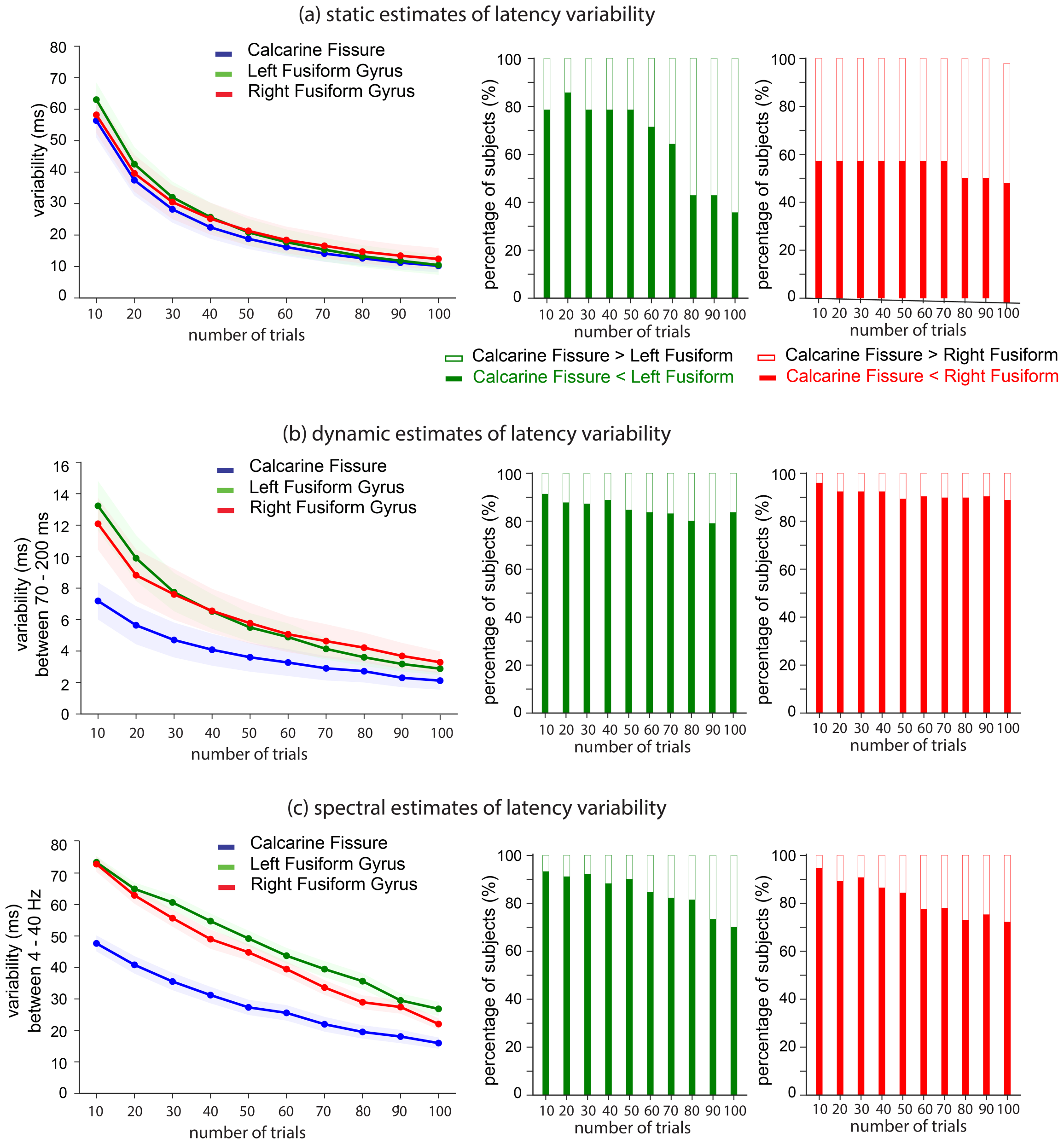

Figure S1. Effects of the number of trials used in one bootstrap sample (*m*) on latency variability (left) and sensitivity to detect the specified sequence of activity (right). (Left) Latency variability across regions when *m* varies from 10 to 100. Shaded areas indicate the standard error of the latency variability across subjects. (Right) Percentage of subjects showing the specified response pattern (variability: calcarine < fusiform) when different numbers of trials are used for one bootstrap sample.
